# Recent Advances in Endoscopy

**Published:** 2017

**Authors:** Dominic King, David Al Dulaimi

**Affiliations:** *Department of Gastroenterology, Alexandra Hospital, Redditch, UK*

***Long Term Outcome of Routine Image-enhanced Endoscopy in Newly Diagnosed Head and Neck Cancer: a Prospective Study of 145 Patients (*** 1 ***)*** ***.***

This prospective, single center study examined the occurrence of synchronous Secondary Primary Tumours (SPTs) of the oesophagus in patients with a new diagnosis of head and neck squamous cell carcinoma (HNSCC). The idea of ‘field cancerization’– where similarly exposed areas to a primary cancer are at higher risk of developing carcinoma – is theorized for the cause of this common phenomenon. The findings of other studies put the incidence of synchronous SPTs in HNSCC as high as 36%. Oesophageal Squamous Cell Neoplasia (ESCN) are the most common of the digestive tract SPTs and if caught early, at a superficial stage, outcomes are good with greater than 80% survival at 5 years.

145 of a total 225 patients were enrolled with a new diagnosis of HNSCC had screening with Image-Enhanced Endoscopy (IEE) of the upper gastro-intestinal (UGI) tract before treatment for the primary tumour. Assessment of the oesophagus, stomach and duodenum was done with white light and the oesophagus was further interrogated under narrow-band imaging with magnifying endoscopy (NBI-ME) and then chromoendoscopy with 2% Lugol’s solution. Biopsies were obtained from 67 patients with lesions satisfying the defined criteria and classified using the revised Vienna classification of epithelial neoplasia. Those with Classification 1-2 (no dysplasia or indeterminate) were followed with 6 monthly IEE and together with low-grade dysplasia (classification 3) made up 123 of the 145 cases. Those with higher grades were reviewed by expert panels to determine future management and 22 of the 145 cases included these higher classifications (4 & 5) and other UGI malignancies. SPTs made up 15.2% of the screened population. Patient characteristics were recorded and analysed for prediction of overall survival using univariate and multivariate analysis. 

The follow up period was from initial primary cancer diagnosis until death. Kaplan-Meier plots and Log-rank tests compared survival of patients with and without SPTs of the UGI tract as well as the stage of primary tumour for those with synchronous oesophageal cancers. 

The study found that those with HNSCC, but without synchronous ESCN had better survival than those with synchronous neoplasia of the UGI tract. Those with later stage concomitant oesophageal neoplasia had the worst survival. There were trends seen, though significance was not reached for patients with advanced HNSCC with early stage ESCN vs those without

The authors conclude that routine IEE screening in new diagnoses of HNSCC is important for prognostication and risk stratification, especially given the early stage at which most of the ESCN were found – where curative treatment remains possible. The authors point to the limitations of this study in that it was a small, single centre study where treatment was not randomised and other comorbidities were not addressed in terms of survival analysis, and they concede that survival benefit was not seen from their screening. Their review of the literature however showed that some longitudinal and cost effectiveness studies have demonstrated that, in particular high risk groups screening can afford survival benefit and further clinical trials are warranted.

***Suppository naproxen reduces incidence and severity of post-endoscopic retrograde cholangiopancreatography pancreatitis: Randomized controlled trial (*** 2 ***).***

This single centre, randomised control trial looked at the prevention of Post endoscopic retrograde cholangiopancreatography (ERCP) Pancreatitic (PEP) using naproxen suppository prior to the procedure. 

PEP is common and the paper references review articles, meta-analysis and a systematic review when describing an incidence of between 1 and 10% and as high as 40% in high risk groups. The inflammatory cascade associated with pancreatic duct manipulation whether through intent or as an unwanted side effect of ERCP is considered here as a target in the prevention of PEP. Several studies on the use of Non-Steroidal Anti-Inflammatory (NSAIDs) agents in this context are cited though not all have shown benefit and some reports have suggested NSAIDs can be a causal agent acute pancreatitis. The best route, dose and type of NSAID are questions that remain unclear.

The inclusion criteria required age over 18, no current or previous history of pancreatitis (chronic or acute), no previous sphincterotomy, no active peptic ulcer disease and no rectal disease. Patients with renal dysfunction, pregnancy/breast feeding, recent NSAID use or hypersensitivity to NSAIDs were also excluded. A total of 324 patients were eligible for a powered study based on the frequency of pancreatitis in the placebo arm vs the treated arm. Patients were randomised and patients, doctors and nurses blinded to the treatment they would receive prior to ERCP. Sedation was either by midazolam or pethidine and 3 experienced practitioners performed the ERCPs.

The study’s main outcome was the occurrence of pancreatitis defined by Cotton et al.^†^ Serum amylase was measured in all patients 2 hours before the procedure and at 24 hours and if upper abdominal pain was complained of. The control group had a mean age of 44.7 ± 9.7 with a slightly higher male population of 54.9%.The naproxen group had a mean age of 46.3 ± 8.3 and 51.9% were male in this arm.

The overall PEP frequency was 12% (40/324) with significantly more in the placebo group 17% vs 7.4%. The authors further analysed the results and describe statistically higher PEP in females, longer ERCP duration and with either pancreatic duct injection or repeated cannulation of the pancreatic duct. However, these latter two subgroups had significantly lower PEP if the patient had received naproxen. All patients were reported to have been discharged well. The diagnoses of patients who developed PEP included choledocholithiasis (9 naproxen / 23 placebo), Sphincter of Oddi Dysfunction (SOD) (1 naproxen/2 placebo), common bile duct tumours (1 naproxen/2 placebo) and choledochal cysts (1 naproxen /1 placebo).

In the discussion the authors point to systematic review and meta-analyses that describes a significant reduction in PEP incidence with the use of NSAIDs. In this study the numbers needed to treat was 10 and though the literature reviewed has NNT ranging from 6.5 to 17. Other studies have also shown that certain patient characteristics play a role in risks associated with PEP. This study in contrast to others uses naproxen as the NSAID of choice and the authors suggest a multicentre study is needed to better understand its usefulness in this area. They conclude that based on this data, pre-ERCP rectal naproxen is a safe and effective preventative of PEP especially in those with risk factors.

***The Association Between Gastric Endoscopic Findings and Histologic Premalignant Lesions in the Iranian Rural Population (*** 3 ***).***

Worldwide, gastric cancer is common. Premalignant Lesions (PML) are precursors to gastric cancer and include atrophic gastritis (AG), intestinal metaplasia (IM) and gastric dysplasia (GD). The paper alludes to the limited knowledge of correlation between what is seen at endoscopy and the histological findings of PML. 

In seeking to review a hypothesised correlation between the macroscopic findings at endoscopy and the histological evidence of PMLs this study examined rural Iranian patients with dyspepsia (defined as epigastric pain or discomfort) over a 28-month period. A single endoscopist performed all procedures on the 1973 patients included in the study. The study population had a mean age of 42.61 (±17.024) with a female preponderance at 65%. 

Each patient had 2 biopsies taken from the antrum and 2 from the gastric body. In addition, any abnormal mucosa was further biopsied. Patients were stratified into three groups: Group 1 had normal macroscopic mucosa and accounted for 55.7% of the population; Group 2 had ulcers with or without concurrent mucosal abnormality (3.8%) and Group 3 had abnormal mucosa but no ulcers and accounted for the remainder at 40.5%. Two expert pathologists then examined the prepared biopsy specimens to give a histological diagnosis and then the groups were compared.

14% of the studied population had evidence of PMLs and statistically significant risk factors included older age, and, in this study, maleness also seems to be a risk factor for abnormal endoscopy and hence PMLs. The prevalence of PMLs in Group 1 was 6.8% (75/1098), Group 2 26.3% (20/76) and Group 3 22.8% (182/799). Though both Groups 2 and 3 showed significantly higher levels of PML compared to group 1 there was no significant difference between groups 2 and 3 (P=0.484). The authors conclude that abnormal endoscopy is a risk factor for PMLs. 

The limitations of the study included the lack of advanced endoscopic techniques, and the authors felt that gastric fundal biopsies would have been appropriate. In discussion the authors point to the literature regarding certain PMLs and the correlation with abnormal endoscopic findings. Although some pathology seems to correlate well endoscopically and histologically (duodenal bulb changes in dyspeptic patients); both in this study and many others, correlation is poor and normal endoscopic findings can still have pathological histology. The authors conclude by stating that based on their findings and current knowledge, abnormal endoscopic findings should be biopsied and in older age, dyspeptic men – normal gastric mucosa at endoscopy should have a random sampling for histological abnormalities. 

***Safety of Digestive Endoscopy following Acute Coronary Syndrome: A Systematic Review (*** 4 ***).***

Endoscopy in the early period after an Acute Coronary Syndrome (ACS) is risky with significant harmful outcomes recorded attributable to the endoscopic procedure. Risk of recurrent ACS, arrhythmias, heart failure and death are raised but a clear agreement on the best time to “scope” in this setting is not apparent in the literature. This systematic review set out to address the current understanding of endoscopy risk in the early period following ACS. 

14 publications were included, from between 1993 and 2014, following an extensive search of MEDLINE, EMBASE, Cochrane library and the ISI Web of Knowledge from between 1990 and 2014. 1178 patients underwent 1188 endoscopies at 9±5.2 days post ACS (10 patients required repeat procedures). Procedures included Oesophago Gastro Duodenography (OGD) (68.2%) and colonoscopy (16.1%), as well as sigmoidoscopy, (Percutaneous Endoscopic Gastroscopy) PEGs and ERCPs (Endoscopic Retrograde Cholangio Pancreatography). Two of the authors classified complications in the publications as either major or minor and the procedure type and indication, timing of events and type of ACS were recorded. The type of sedation used was not specified and not all of the publications reviewed reported the use of sedation, however 87.0% (95% CI 84.2–89.3%) of patients received some form of sedation. The literature suggests that the type of sedation used 

Indications were classified as Haematemesis (14.2%), Malaena (12.7%), Bright Red Blood Per Rectum (5.3%), Gastrointestinal Bleeding (28.8%) Occult Bleeding (20.39%), and other (18.8%). Therapy was performed in 20.2%though the type was not specified but included PEG procedures, 

From all the endoscopy types within the review, complications (including death) occurred in 9.1% of cases (95% CI 7.6-10.9%) of which 72.4% were classified as “minor”. There were 4 deaths which occurred within 24 hours of endoscopy 3 were due to arrhythmias during the endoscopic procedure and they account for 3.7% (CI 1.5-9.1%) of complication rates. All-cause mortality was found to be 8.1% (95% CI 6.2-10.1%). Where specified (44.4% complications not specified) it was hypotension, arrhythmias and recurrent ACS which accounted for most of the complications associated with endoscopy in the period after initial ACS.

The paper concedes several limitations – in particular selection bias associated with publications having had the same author (though duplicate data was excluded) and the poor quality, heterogeneity of the data. 

The authors emphasise the risk of complications associated with endoscopy in the context of recent ACS – 11.5% (95% CI 9.2–14.4%) for OGD and 9.0%(95% CI 4.8–16.2%), for colonoscopy seen in this study – compared to 1 in 200 to 1 in 10,000 seen in elective OGDs and 2.8 in 1000 for screening colonoscopies. The authors suggest that the increase in percutaneous intervention and associated antiplatelet and anticoagulant agents will make GI bleeding more common, such that if preventative gastro-protection is unsuccessful, better endoscopic techniques to improve outcomes are required. They further advise increased cardiovascular monitoring in this setting and careful consideration of the risks and benefits likely to be accrued by performing endoscopy in this population.

## References

[1] Chung C-S, Lo W-C, Wen M-H, Hsieh C-H, Lin Y-C, Liao L-J (2016). Long Term outcome of routine image-enhanced endoscopy in newly diagnosed head and neck cancer: a prospective study of 145 patients. Sci Rep.

[2] Mansour-Ghanaei F, Joukar F, Taherzadeh Z, Sokhanvar H, Hasandokht T (2016). Suppository naproxen reduces incidence and severity of post-endoscopic retrograde cholangiopancreatography pancreatitis: Randomized controlled trial. World J Gastroenterol.

[3] Niknam R, Manafi A, Fattahi MR, Mahmoudi L (2015). The association between gastric endoscopic findings and histologic premalignant lesions in the Iranian rural population. Medicine (Baltimore).

[4] Dorreen A, Moosavi S, Martel M, Barkun AN (2016). Safety of digestive endoscopy following acute coronary syndrome: a systematic review. Can J Gastroenterol Hepatol.

